# Effects of different re-warm up activities in football players' performance

**DOI:** 10.1371/journal.pone.0180152

**Published:** 2017-06-29

**Authors:** Eduardo Abade, Jaime Sampaio, Bruno Gonçalves, Jorge Baptista, Alberto Alves, João Viana

**Affiliations:** 1 Research Center in Sports Sciences, Health Sciences and Human Development, CIDESD, Portugal; 2 University Institute of Maia, ISMAI, Maia, Portugal; 3 University of Trás-os-Montes e Alto Douro, UTAD, Vila Real, Portugal; Universita degli Studi di Verona, ITALY

## Abstract

Warm up routines are commonly used to optimize football performance and prevent injuries. Yet, official pre-match protocols may require players to passively rest for approximately 10 to 15 minutes between the warm up and the beginning of the match. Therefore, the aim of this study was to explore the effect of different re-warm up activities on the physical performance of football players. Twenty-Two Portuguese elite under-19 football players participated in the study conducted during the competitive season. Different re-warm up protocols were performed 6 minutes after the same standardized warm up in 4 consecutive days in a crossover controlled approach: without, eccentric, plyometric and repeated changes of direction. Vertical jump and Sprint performances were tested immediately after warm up and 12 minutes after warm up. Results showed that repeated changes of direction and plyometrics presented beneficial effects to jump and sprint. Different practical implications may be taken from the eccentric protocol since a vertical jump impairment was observed, suggesting a possibly harmful effect. The absence of re-warm up activities may be detrimental to players’ physical performance. However, the inclusion of re-warm up prior to match is a complex issue, since the manipulation of volume, intensity and recovery may positively or negatively affect the subsequent performance. In fact, this exploratory study shows that eccentric exercise may be harmful for physical performance when performed prior a football match. However, plyometric and repeated changes of direction exercises seem to be simple, quick and efficient activities to attenuate losses in vertical jump and sprint capacity after warm up. Coaches should aim to develop individual optimal exercise modes in order to optimize physical performance after re warm activities.

## Introduction

Warm up (WU) routines prior to exercise are widely explored by literature and commonly accepted for optimizing performance. Such routines include short-duration and high-intensity activities that intend to enhance physical predisposition by increasing intra-muscular temperature, nerve conductance rate and metabolic reactions [1]. In fact, it is well documented that muscle performance can be acutely improved following a preloading maximal or near maximal stimulus due to the induction of post-activation potentiation [2]. Although physiological readiness and injury prevention seem to be consensual outcomes of the WU, its structure and protocols used are still controversial. Moreover, there is still a disconnection between evidence-based methods and on-field practices.

Other hot topic is the search for the best combination of volume, intensity and recovery, particularly between the WU and subsequent exercise. Under this scope, coaches should be aware that if the WU protocol is too intense or does not allow adequate recovery, post-activation potentiation and consequent performance may be compromised, and on the other hand, if too much time passes, post activation potentiation effects can be lost [3]. It has already been showed that moderate rest periods durations (7–10 minutes) may enhance power output after a conditioning activity, although this trend is highly dependent on the intensity, volume of the conditioning exercise and the individuals’ training status [3]. Thus, adding up to the high inter variability response to the conditioning activity, the selection of the most adequate variables to manipulate post-activation potential remains inconclusive. Taking this in consideration, the effectiveness of a WU also relies on the re-warm up (R-WU) activities implemented during the period between the end of the WU and the start of competition. However, few studies have examined the effects of specific post-activation activities on footballers and have exclusively focused on the R-WU during half-time [4]. These have shown that passive half-times appear to impair sprint and jump performance during the initial phase of the second half whereas R-WU effectively attenuates such deteriorations [5].

Traditionally, official pre-match protocols allow players to return to the changing room after the WU. The time-course activities performed during this period traditionally include equipment/medical concerns and coach feedbacks. In international competitions (e.g. Champions League), teams’ presentation and official anthems imply that players may passively rest for approximately 15 minutes before the match. As a result, the WU short-term effects that are highly dependent on alterations in muscle temperature may be lost [4]. In fact, it was already demonstrated that every 1°C reduction in muscle temperature corresponded to a 3% reduction in lower-body power output [6]. Consequently, decreases in muscle temperature arising from passive periods may contribute to the deterioration of performance, such as the decrease of the jump or sprint capacity. The negative impact of passive periods in performance reinforces the importance of using passive and active R-WU activities aiming to attenuate losses in body temperature [1]. Within the passive activities, heated clothing and heating pads are considered efficient methods to attenuate body temperature loss and avoid significant decreases in performance such as in repeated sprints [7]. However, active R-WU may also benefit subsequent physical performances [8].

As previously mentioned, strength exercises for problematic areas, plyometrics and agility are common procedures used by football coaches during WU to enhance performance and prevent injuries. However, little is known if these are adequate activities to induce positive acute short-term effects in the time frame between the end of WU and the beginning of the match. It has been shown that hamstrings exercises such as Nordic may induce medium-term improvements of hamstrings strength and dynamic control ratios in soccer players [9]. However, unaccustomed eccentric muscle activity may result in significant damage to contractile and structural components of skeletal muscle [10]. The plyometric movements are capable of eliciting muscular strength potentiation, which may improve power and dynamic performance in stretch-shortening cycle movements such as sprinting and jumping actions [11]. The change of direction is an important skill in football because it reproduces intermittent and multilateral high speed movements performed during the match with a high physiological impact [12, 13].

Even though players are commonly grouped during WU routines to perform the previously mentioned strategies (i.e. plyometrics, changes of direction and strength exercises), individual post-activation responses may emerge from different conditioning activities, which demands for an accurate analysis in terms of the optimal recovery time. Therefore, the aim of this study was to explore the effect of different R-WU activities performed immediately before the match in the physical performance of football players. It is anticipated that: 1) plyometrics will have a positive acute impact in vertical jump; 2) changes of direction exercises will positively affect the sprint capacity; and 3) eccentric exercise will be detrimental for both jump and sprint capacities.

## Materials and methods

### Participants

Twenty-two elite male under-19 soccer players (age: 18.3 ± 0.5 years old, ranging from 17 to 19 years old; stature: 181.7 ± 6.4 cm; body mass: 73.6 ± 5.8 kg; playing experience: 9.6±1.8 years) from the same team competing in the Portuguese national championship participated in this study. Participants were recruited 30 days before the beginning of the study. At the time that the study was conducted, players performed from 4 to 5 training units a week, ~90 minutes per session, with an official match during the weekend. Written informed consent was obtained from all participants and their parents. Despite this, they were free to withdraw from the study at any time without any penalty. All procedures were in accordance with the ethical standards of Ethics committee of Research Center in Sport, Health and Human Development (UID/DTP/04045/2013) and with the 1964 Helsinki declaration.

### Design

A crossover controlled study was conducted during the competitive season. 4 protocols (WU + R-WU) were tested in 4 consecutive days in a randomized sequence, i.e., all players were submitted to the same randomized protocol in each day. The protocols had identical durations and differed only in the re-WU strategy applied after the standardized WU: without R-WU (CON), eccentric R-WU (ECC), plyometric R-WU (PLY) and repeated changes of direction R-WU (RCOD).

### Methodology

Evaluations were performed at the same time of the day (15:00 PM) on artificial turf pitches, under similar environmental conditions (temperature 18–25°C, relative humidity 48–56%). The standard active WU consisted of a brief aerobic portion, dynamic stretching, post-activation exercises and sprints/race-pace efforts [14] structured as following: 2 minutes jogging and 4x30m side run; dynamic stretching for main locomotive lower limb muscles [15] (2x10 hip adduction, 2x10 hip abduction, 2x10 butt kicks, 2x10 knee raises and 2x10 straight leg march), dynamic strength exercises (2x10 deep squats, and 2x10 forward lunges); incremental intermittent sprints and agility runs as follow: 2x10m ¾ pace, 2x20m ¾ pace, 2x30m ¾ pace, 1x30m with 10 decelerations (both feet), 1x30m with 10 decelerations (one foot), 2x30m with 4 changes of direction ¾ pace (90°), 1x20m full pace and 1x30m full pace. The total time of the WU was approximately 15 minutes.

**Fig 1:**
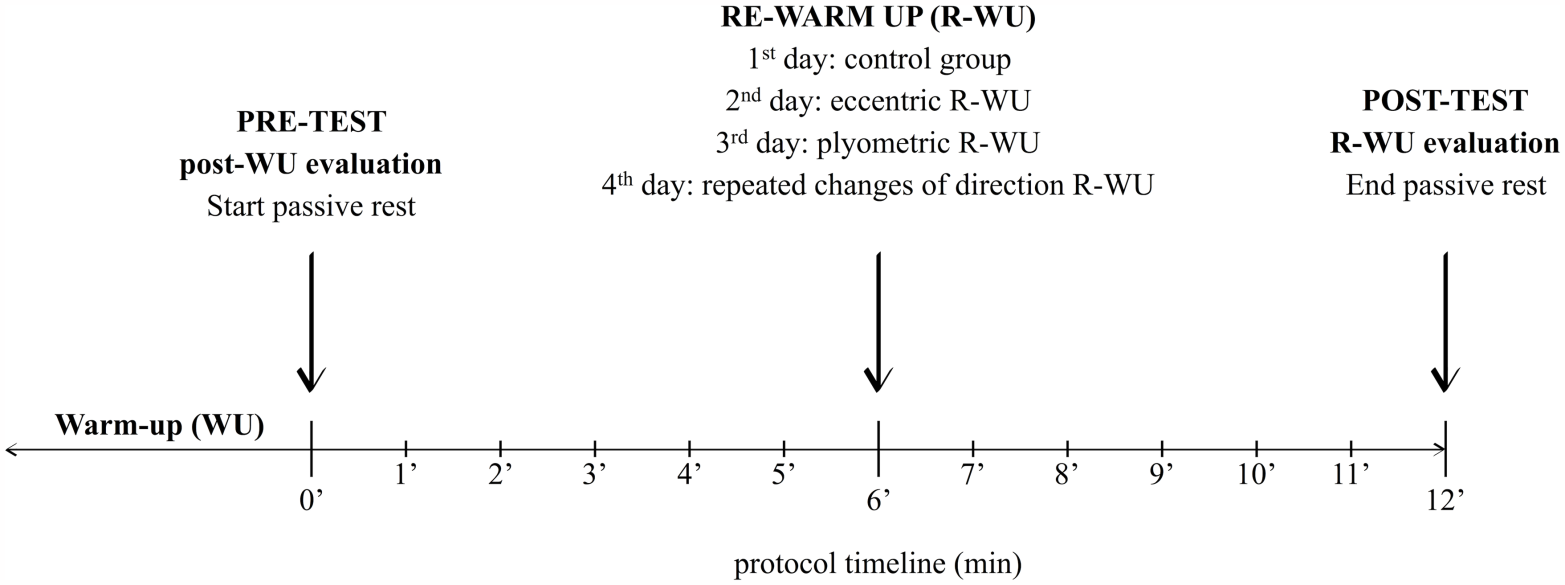
Schematic representation of experimental design. The both Post-WU (immediately after WU) and R-WU (12 minutes after WU) evaluations tests were included to assess differences in R-WU protocols. The R-WU activities were completed in randomized sequence across 4 days.

After the WU, the CON group passively rest for 12 minutes, while the other groups performed eccentric, plyometrics or repeated changes of directions tasks exactly 6 minutes after WU (see Fig 1). The ECC players were asked to perform the Nordic Hamstring exercise [16] in 4 sets of 3 reps with 15 seconds of passive recovery between sets. Players were instructed to place the hands on the chest and to gradually lean forward while resisting during 3 seconds keeping the trunk and hips in a neutral position throughout the whole range of motion. The PLY group performed 4 sets of 5 jumps over 40 cm barriers (hips, knees and ankles bended until the knees were flexed to 90 degrees) followed by one foot runs in an agility ladder with 10 rungs. A 15 seconds’ passive recovery was allowed between sets. Finally, the RCOD group performed 4x20m sprints with 4 changes of direction (100°) [17] with 15 seconds of passive recovery. Despite the natural different mechanical demands inherent to the different activities, R-WU exercises had similar durations in order to guarantee a coherent inclusion of such activities in the time gap between the end of the WU and the beginning of the match. None of the players were familiarized with the R-WU protocols.

Explosive actions such as jumping and sprinting are important contributors to football performance [18]. Vertical jump is recognized as a useful index of the muscular ability to generate power and can be used to monitor performance as well as to provide important information about the functional ability of lower limbs under different conditions [19]. Counter movement jump (CMJ) and Abalakov jump (AJ) were used to assess the VJ capacity [20] with an Optojump device (Microgate, Italy). To perform the CMJ, participants were asked to stand upright, place their hands on the hips and stay there throughout the test. When ready, the athlete squatted down until the knees were bent at 90 degrees, then immediately jumped vertically as high as possible. To perform AJ subjects were allowed to swing the arms back behind when bending the knees and forward while jumping vertically.

Subjects performed 2 maximal attempts at each VJ exercise with the best result being recorded for analysis. A 30-second interval was allowed between the 4 jumps [21]. High intensity runs and sprinting may represent approximately 10% of the total distance covered during a match [22]. As they are mostly performed in shorter distances (10-30m) [23], evaluating players under these conditions may represent an important potential indicator. The sprint time for 10-m and 20-m was measured using an infrared timing system with three photo cells (Witty, Microgate, Italy), allowing both times to be recorded in just one attempt. Participants were asked to assume a stationary start position with the front foot placed 50 cm behind the first cell. Sprint tests were performed 30 seconds after the VJ assessment. All previous tests were performed immediately after WU (pre-test, post-WU evaluation) and 12 minutes after WU (post-test, R-WU evaluation) (see Fig 1). This time gap was established according to the average time interval usually reported by coaches that have as a reference the official U-19 pre-match protocol (10 to 15 minutes). Players were evaluated in a sequentially mode so to ensure that the time from the R-WU routine to the assessment was identical for all players.

### Statistical analysis

Individual and mean changes from post-WU to R-WU for all considered R-WU activities were graphically represented and the variation from considered moments expressed in percentage variation (mean±SD). Also, both intra-day (between-players) and day-to-day variability (within-players) in post-WU performance was measured as typical error and expressed as a coefficient of variation, CV (%) [24]. To realize the possibly beneficial/harmful effects of between-R-WU interventions on players’ performance measures, the data were analysed with a specific spreadsheet for pre-post crossover trial [25]. The effects were estimated in percent units through log-transformation and uncertainty in the estimate was expressed as 90% confidence limits. The outcome for performance measures was evaluated with the clinical/practical version of magnitude-based inference: suggested default probabilities for declaring an effect clinically beneficial are <0.5% (most unlikely) for harm and >25% (possibly) for benefit; a clinically unclear effect is therefore possibly beneficial (>25%) with an unacceptable risk of harm (>0.5%) [26]. Probabilities were reported using the following scale: 25–75%, possibly; 75–95%, likely; 95–99.5%, very likely; >99.5%, most likely. Standardized (Cohen) mean differences, and respective 90% confidence intervals were also computed as magnitude of observed effects, and, thresholds were 0.2, trivial; 0.6, small; 1.2, moderate; 2.0, large; and >2.0, very large [26].

## Results

**Fig 2:**
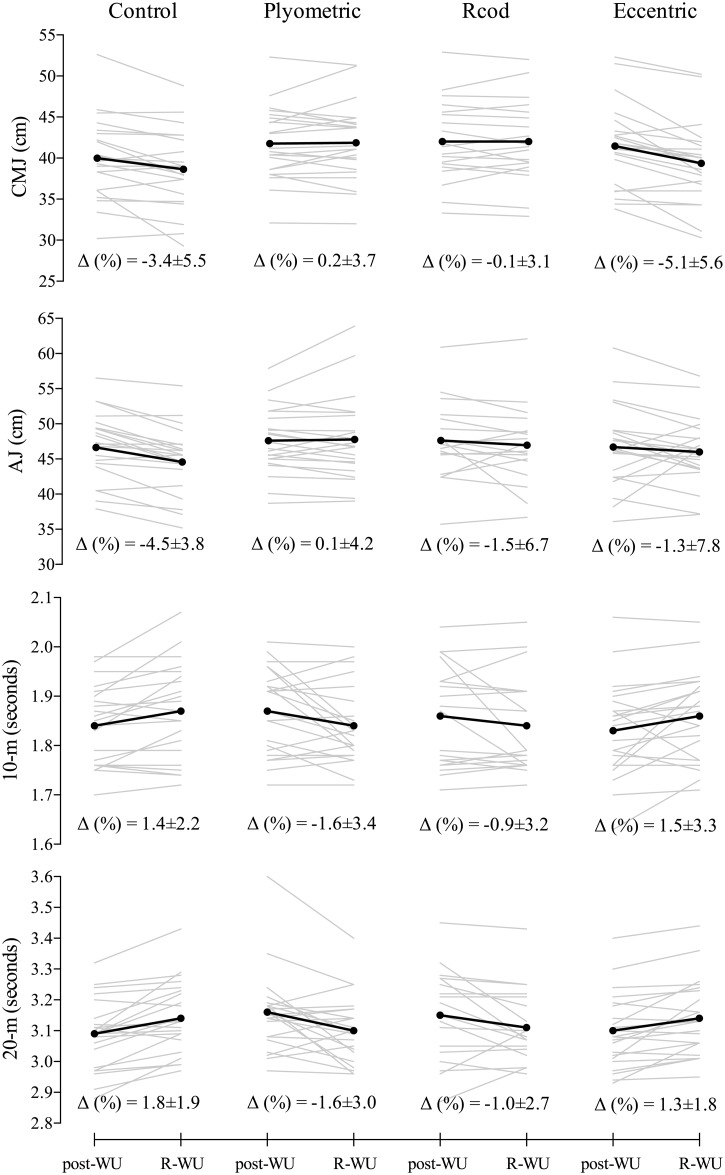
Individual and mean changes from post-WU to R-WU for counter movement jump (CMJ), Abalakov jump (AJ), 10-m and 20-m- sprint. Percentage variations (Δ%) are expressed as mean±std. Black lines represents the group mean changes while grey represents individual changes.

**Fig 3:**
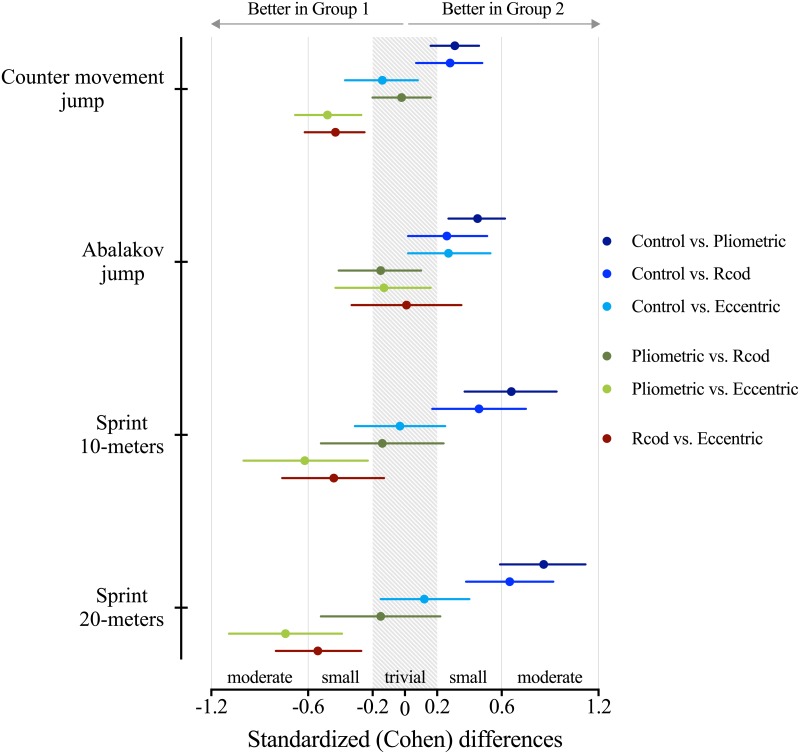
Standardised Cohen’s differences for counter movement jump (CMJ), Abalakov jump (AJ), 10-m and 20-m- sprint according to R-WU activities comparisons. Error bars indicate uncertainty in true mean changes with 90% confidence intervals. Note: since lower time in sprint protocols are related with better performance, the outcomes for 10-m and 20-m were changed from negative to positive, and vice-versa. This decision was made for a better interpretation of the results.

Individual and mean changes from post-WU to R-WU in considered performance measures are shown in Fig 2. Complementary, Fig 3 and Table 1 presents the standardized differences and practical inferences, respectively, based on the pre-post crossover trial analysis.

**Table 1:** Inferences for the re-WU interventions on players’ performance measures.

Variables	Groups	Group comparison outcomes as: Mean changes (%; ±90%CL) % Chances (harmful/trivial/beneficial) Practical inferences		
		Plyometric	Rcod	Eccentric
CMJ	Control	3.8; ±1.8	3.5; ±2.6	-1.8; ±2.7
		0/10/90	0/25/75	35/65/1
		likely beneficial	likely beneficial	possibly harmful
	Plyometric	-	-0.3; ±2.0	-5.3; ±2.2
		-	6/92/2	99/1/0
		-	likely trivial	very likely harmful
	Rcod	-	-	-5.1; ±2.1
		-	-	98/2/0
		-	-	very likely harmful
AJ	Control	4.8; ±1.9	3.1; ±2.9	3.2; ±3.1
		0/1/99	0/31/69	0/30/70
		very likely beneficial	possibly beneficial	possibly beneficial
	Plyometric	-	-1.6; ±2.7	-1.5; ±3.3
		-	38/61/1	36/61/1
		-	possibly harmful	possibly harmful
	Rcod	-	-	0.1; ±4.2
		-	-	15/67/18
		-	-	possibly trivial
Sprint 10-meters	Control	-2.9; ±1.3	-2.3; ±1.4	0.1; ±1.3
		0/1/99	0/6/94	15/76/9
		-very likely beneficial	likely beneficial	likely trivial
	Plyometric	-	0.7; ±2.0	3.2; ±2.0
		-	40/53/7	96/4/0
		-	possibly harmful	very likely harmful
	Rcod	-	-	2.5; ±1.8
		-	-	likely harmful
		-	-	likely harmful
Sprint 20-meters	Control	-3.4; ±1.0	-2.8; ±1.1	-0.5; ±1.0
		0/0/100	0/0/100	3/64/33
		most likely beneficial	most likely beneficial	unclear
	Plyometric	-	0.7; ±1.6	3.0; ±1.5
		-	42/52/6	99/1/0
		-	possibly harmful	very likely harmful
	Rcod	-	-	2.4; ±1.2
		-	-	98/2/0
		-	-	very likely harmful

Note: 90% CL = 90% confidence limits. CMJ = counter movement jump. AJ = Abalakov jump.

The intra-day (between-players) post-WU players’ performance presented ~11% variation in jumps (CV %, mean±SD, 11.7±0.7 in CMJ and 11.3±1.2 in AJ) and ~4.5% variation in sprints (4.9±0.6 in 10-m and 4.1±0.4 in 20-m). On the other hand, inter-day (within-players) post-WU players’ performance presented ~4% variation in jumps (4.2±2.6 in CMJ and 4.1±2.6 in AJ) and ~3.5% variation in sprints (3.2±1.7 in 10-m and 2.3±1.1 in 20-m).

There were small-to-large beneficial improvements in all performance measures when player underwent PLY intervention (Table 1). Both CMJ and AJ showed a likely/very likely improvement (~3.8% in CMJ and ~4.8% in AJ) when compared to the CON group (i.e., same players without R-WU intervention scenario). The same R-WU intervention showed a moderate very likely beneficial effect in 10-m (ES = Cohen d; ±90%CL, -0.7; ±0.3) and a moderate most likely beneficial effect in 20-m (ES = -0.9; ±0.3). Individual changes from post-WU to R-WU showed a 0.2±3.7% variation in CMJ, 0.1±4.2% in AJ, while CON presented a -3.5±5.5% in CMJ and -4.5±3.8% in AJ (Fig 2). Also, both 10-m and 20-m resulted in a change of -1.6±3.4% and -1.6±3.0% variation in sprint duration, respectively, while CON showed an increase of 1.4±2.2% in 10-m and 1.8±1.9% in 20-m.

The RCOD presented slightly similar effect on players’ performances to the PLY. Main differences were well related to the individual variability changes from post-WU to R-WU performance since players presented a decrease of ~1% in jump height performances and an increase of ~1% in sprints duration (Figs 2 and 3). However, the R-WU intervention presented a likely/possibly beneficial effect to CMJ (ES = 0.3; ±0.2) and AJ (ES = 0.3; ±0.2), respectively, and a moderate likely/most likely beneficial effect to 10-m and 20-m sprint performances when compared to CON group. When compared to PLY intervention, the RCOD presented a possibly harmful effect in AJ, 10-m and 20-m performance tests (trivial effect sizes) (see Table 1 and Fig 3).

Different practical implications may be taken from the ECC R-WU intervention. There was a -5.1±5.6% decrease in CMJ height from post-WU to R-WU which affords a possibly harmful effect through such R-WU application when compared to CON (35% chance of being harmful, 65% trivial and 1% beneficial) (Fig 2 and Table 1). Also, regarding 10-m and 20-m sprint, the considered R-WU presented likely trivial and unclear effects, respectively. The exception was identified in AJ performance, since 70% of players possibly benefited from it (Table 1). Finally, compared to both PLY and RCOD interventions, the ECC presented from possibly to very likely harmful effect in CMJ, 10-m and 20-m sprint performance (from small to moderate effect) (Table 1 and Fig 3).

## Discussion

This study shows that the absence of R-WU activities in the time gap between the end of the WU and the beginning of the match may impair the players’ physical performance. While performing eccentric exercise before a football match may be even harmful, losses in power output are attenuated by performing active R-WU exercises such as plyometrics and repeated changes of direction. In fact, the PLY and RCOD interventions tested in this study showed to be important acute post activation potentiation activities in both vertical jump and sprint capacity. This may be due to the fact that different and specific post activation potentiation regimens may optimize related functional tasks by improving performances in similar force-orientation production [27].

Short-duration high-intensity contractions are known to enhance subsequent explosive muscular performance by increasing central nervous system activity. This neural phenomenon was also hypothesized to occur when high intensity plyometrics precede maximal muscle strength exercises. In fact, plyometrics may elicit muscular strength potentiation which improves power output, explosiveness and dynamic performance [11]. Apparently, short-duration high-intensity exercises such as drop jumps may increase the recruitment of high-threshold motor units, contributing to enhance subsequent explosive exercises [28]. In fact, it was already reported an acute increase in the athletes’ ability to apply greater levels of force against the ground after vertical drop jumps tasks which supports the importance of vertical ground reaction forces as a key kinetic component for vertical explosive actions [27]. Also, horizontal actions seem to benefit from plyometrics post activation activities, partly because these exercises have a great biomechanical specificity (e.g. ground contact times) to sprinting [29]. In the present study, performing 4 sets of 5 jumps followed by bilateral skipping was effective in attenuating losses in jump and sprint capacities in the time course between the end of WU and beginning of the match. These results show that sprint can also be acutely improved by the performance of jumping exercises. The fact that the PLY intervention included both vertical (i.e. drop jumps) and horizontal (i.e. bilateral skippings) oriented exercises, seems to confirm that specific post activation potentiation exercises induce acute performance enhancements in similar force-orientation tasks (see Fig 3).

The ability to perform changes of direction (COD) while running is an important factor for successful performance in team sports. It was already showed that COD enhance lower limb muscle activity when compared with linear sprints [30], which may be related to the substantial lateral forces applied on the ground [31]. Moreover, 90° COD sprints seem to elicit greater EMG muscle activity when compared to lower angle COD such as 45°, probably due to the highest mechanical demands of deceleration and re-acceleration patterns [13]. As the present investigation used R-WU RCOD activities with 100° angles, it is assumed that kinematic and metabolic demands of this task elicited similar potentiation effects as observed in PLY, with short duration high intensity COD contributing to enhance subsequent explosive performance in vertical and horizontal oriented tasks (see Fig 3). As this R-WU strategy was applied 6 minutes after WU and lasted for approximately 2 minutes, it appears that the balance between muscular activation and recovery time resulted in performance potentiation. In fact, optimal performance occurs when fatigue is overcome and potentiated effect still exists [32].

Because lower levels of hamstring strength are suggested to increase the risk of hamstring injuries [33], Nordic Hamstring has been proposed as an important exercise to increase eccentric hamstring muscle strength and decrease the injury likelihood in football players. However, familiarization and training load history may influence the acute effects of Nordic in physical performance. For that reason, the injury prevention FIFA 11+ program suggests a different number of sets and repetitions for novice, intermediate and expert players [34]. Generally, eccentric exercise induces muscle damage derived from overstretching and disrupted sarcomeres, which may acutely impair muscle voluntary contraction force [35]. Also, the length—tension relation of the muscle may be altered during eccentric exercise [35]. For example, Nordic exercise implies alterations in the length-tension relationship of hamstrings, with a shift in the angle of peak torque to longer lengths [36]. As fatigue may occur due to mechanical detrimental effects related to the disruption of the myofibrillar architecture and since muscle damage is proportional to previous contractile intensity [37], it is assumed that Nordic exercise leads to a greater mechanical trauma, which would require a longer recovery phase to hypothetically induce positive effects over time. All these neuromuscular acute adaptations to eccentric exercise resulted in an impairment of the explosive muscle performance as showed by the harmful effect of this R-WU strategy on the vertical jump and sprint capacities. Interestingly, AJ was not severely impaired, which might indicate that the use of arm swinging represent an important factor to overcome the negative acute effects induced by the eccentric exercise.

Re-activation interventions have been exclusively investigated during half-times and it seems consensual that R-WU is superior to no R-WU in maintaining or improving physical performance. However, until this moment no data were available to clarify if R-WU activities are also valid in the time gap between the end of the WU and the beginning of the match. This study shows that PLY and RCOD are simple and practical post activation activities that induce acute positive effects in both vertical jump and sprint capacities. Nevertheless, coaches should be aware that players may experience dissimilar levels of fatigue after R-WU interventions and that individual acute responses may emerge from the same post activation exercise. One limitation of this study is that only the acute effects of the R-WU were considered. For that reason, future studies should investigate the time-course of the effects of different R-WU activities in the internal and external workload profiles of football players.

## Practical applications

This study explored the acute effects of R-WU performed in the time gap between the end of the WU and the beginning of the match. The major findings were:

- R-WU exercises such as plyometrics and repeated changes of direction are simple, quick and efficient activities to attenuate losses in power output during vertical jump and sprint activities after WU.
- Eccentric exercises like the Nordic Hamstrings may be harmful to physical performance when performed immediately before a football match, particularly if players are not familiarized with it.
- Coaches should be aware of a great inter-variability in the response of different R-WU activities, which reinforces the need of individual prescription.

## Conclusions

The mode of the conditioning exercise, intensity, recovery time, type of contraction as well as the players’ training status will determine an individual response in either potentiating a subsequent exercise task or inducing fatigue [38, 39]. Under this scope, research suggests that, generally, post activation potentiation mechanisms WU effects are more efficient in well trained individuals rather than in recreational athletes, probably due to their capacity to recruit more motor units at a higher firing rate during the conditioning exercise [40]. For this reason, coaches should determine each individual’s optimal exercise mode in order to optimize the positive effect of the R-WU activity in players’ performance response after the WU. PLY and RCOD are efficient post activation activities that induce acute positive effects in both vertical jump and sprint capacities. Future investigations should be focused on the effects that different R-WU activities may have across the 90 minutes of a competitive match.
